# Genome-wide scans for footprints of natural selection

**DOI:** 10.1098/rstb.2009.0219

**Published:** 2010-01-12

**Authors:** Taras K. Oleksyk, Michael W. Smith, Stephen J. O'Brien

**Affiliations:** 1 Biology Department, University of Puerto Rico at Mayaguez, Mayaguez 00681, Puerto Rico; 2 Laboratory of Genomic Diversity, National Cancer Institute at Frederick, Frederick, MD 21702, USA; 3 Core Genotyping Facility, Advanced Technology Program, SAIC-Frederick, National Cancer Institute at Frederick, Frederick, MD 21702, USA

**Keywords:** genomes, genome-wide selection scans, whole genome sequences, candidate genes, human populations, vertebrate species

## Abstract

Detecting recent selected ‘genomic footprints’ applies directly to the discovery of disease genes and in the imputation of the formative events that molded modern population genetic structure. The imprints of historic selection/adaptation episodes left in human and animal genomes allow one to interpret modern and ancestral gene origins and modifications. Current approaches to reveal selected regions applied in genome-wide selection scans (GWSSs) fall into eight principal categories: (I) phylogenetic footprinting, (II) detecting increased rates of functional mutations, (III) evaluating divergence versus polymorphism, (IV) detecting extended segments of linkage disequilibrium, (V) evaluating local reduction in genetic variation, (VI) detecting changes in the shape of the frequency distribution (spectrum) of genetic variation, (VII) assessing differentiating between populations (*F*_ST_), and (VIII) detecting excess or decrease in admixture contribution from one population. Here, we review and compare these approaches using available human genome-wide datasets to provide independent verification (or not) of regions found by different methods and using different populations. The lessons learned from GWSSs will be applied to identify genome signatures of historic selective pressures on genes and gene regions in other species with emerging genome sequences. This would offer considerable potential for genome annotation in functional, developmental and evolutionary contexts.

## 1. Introduction

Celebrating the 350th anniversary of the Royal Society, and perhaps more importantly the beginning of recorded publication of science, reminds us that discerning the reason and rationale for biological activities is an ancient though honourable and cumulative process. As the science giants atop whose shoulders we gaze to the future imputed from observations, empiricism and reasoning, today our students face a deluge of digital DNA sequence information, more than we can absorb or interpret very competently. Yet, while our scientific forefathers forged new approaches through deduction, today's genomics scientists mine sequence patterns and perturbations with numerical approaches and computational algorithms. The evolutionary paradigm of adaptation by natural selection of endemic gene variation among individuals is also celebrating an anniversary—150 years since Charles Darwin published the timeless ‘On the Origin of Species’. In this chapter, we shall look forward from a time now when a few dozen mammal species enjoy a published whole genome sequence after the first, human, was deposited in a public database in 2001 (Lander *et al*. 2001). We are slowly learning the exercise of annotating a genome sequence—identifying genes, paralogues, repeats, single nucleotide polymorphisms (SNPs), gene synteny, micro-RNAs, transcriptome, extended haplotypes and other genome features. Geneticists are learning to resolve the functionality, history and beginnings of genome patterning, but we still have much to learn. Here, we explore the sequence motifs and variances that evolutionary experts have proposed and applied to uncover evidence of historic selection in populations, notably humankind.

Genomic variation develops from a combination of evolutionary influences that consist of successes and failures of genes on a backdrop of neutral variation shaped by genome instability, mutation process and demographic history. In truth, a challenge of genome analysis is to determine whether patterns of nucleotide variation can be explained by random drift versus selection pressures. Aspects of selection signatures depend on type, age and strength of selection events. Natural selection acts in at least three modes: positive, purifying (also called stabilizing or negative, eliminating a damaging allele) and balancing selection (including heterozygote advantage and frequency-dependent selection). Each of these selection modes is a response to the external pressure, and each operates to change allele frequencies; yet, each leaves a specific mark on genome variation and architecture. For instance, positive selection decreases genetic variation by favouring an advantageous allele, while purifying selection maintains the integrity of functional sequences by eliminating deleterious mutations. In contrast, balancing selection acts to maintain polymorphism: overdominant selection favours heterozygotes, while frequency-dependent selection and selection in local environments can cause different alleles to be favoured in different localities, and at different times. Discerning selective signatures can become complicated when alternate selection modes act upon the same chromosomal regions, simultaneously or during distinct periods of a population's evolutionary history.

**Figure 1.**
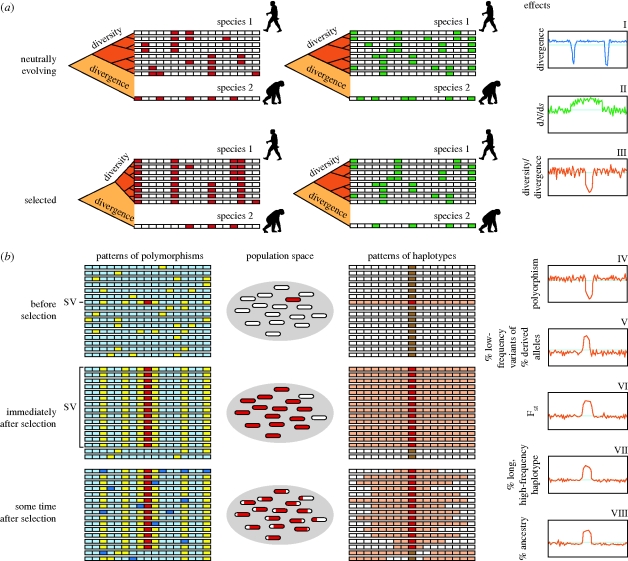
Strategies for detection of the genome-wide selection signatures in table 1. Consider a small gene region that displays SNP variation at 17 adjacent sites (vertical columns in all panels). (*a*) Eight individuals in species 1 (human) carry alternative white and green alleles (synonymous variants) and also a codon-altering non-synonymous allele (red and white). A related species (chimpanzee), examined at the same SNP sites, displays a divergence pattern from the index (human species); positive selection of one SNP allele alters the random distribution pattern when examining non-synonymous alleles only (red and white). Graphs on right plot departure of genome-wide average for parameter (measured by the seven selection tests described in table 1). (*a*) Comparing sequence divergence between species (table 1, I–III). Gene regions with past actions of selection show an altered sequence organization that can be revealed by comparing changes between homologous sequences by three different approaches. (I) Phylogenetic shadowing: comparing divergence of orthologous sequences across the genome. The genome segments with low divergence between species compared with the genome-wide averages can indicate purifying selection or positive selection. (II) Increased function-altering mutation rates: comparing the ratio of non-synonymous (d*N*: left panel; changes indicated in red) to synonymous changes (d*S*: right panel; changes in green). This comparison could be accomplished by (i) comparing the d*N*/d*S* ratio between the candidate gene of interest and the genome-wide average for other genes and (ii) comparing diversity with divergence ratio for d*N* versus d*S* for homologous sequences. (III) Interspecies divergence versus intraspecies polymorphism: comparing intraspecific divergence (e.g. between chimpanzee and human) with interspecific polymorphism (within the human species). Selection decreases variation within an affected species (dark orange), and the scope of this decrease can be assessed by contrasting with divergence between species sequences (light orange) unaffected by the species-specific adaptation. (*b*) Comparing sequence variation patterns within a species (table 1, IV–VIII). Positive selection results in an elevated frequency of haplotypes carrying the advantageous allele at the expense of the others in the process called ‘selective sweep’ (Maynard Smith & Haigh 1974), followed by the gradual incorporation of derived variation seen as a skewed ‘frequency spectrum’. These signatures can all be revealed by comparing sequences within or between populations of the same species. Five tests (described in table 1) include: (IV) Local reduction in genetic variation: comparison of levels of polymorphism in and around the selected locus to the estimated neutral expectation or to the genome-wide averages (left panel; ancestral alleles are in blue or light blue). (V) Changes in the shape of the frequency distribution: identifying an excess of derived alleles, low-frequency polymorphic sites or singletons. Generations after the selective sweep, new (derived) mutations (yellow) are slowly introduced back into the recently selected region, and most appear at low frequencies expected under mutation/drift equilibrium, resulting in a skewed frequency distribution (spectrum) of polymorphisms (left panel). (VI) Differentiating between populations: identifying regions of unusually high population divergence. Local reduction of genomic variation in a selected population (left panel, middle) results in a local increase in genomic differentiation between sequences (unaffected population is not shown in the figure but can be approximated by the population before selection: left panel, top). Comparisons can be made for levels of differentiation calculated as *F*_ST_ around the selected loci to the neutral expectations, to a set of neutral loci or to the genome-wide averages. (VII) Extended LD segments: comparing the relative length and frequency of selected haplotypes. Positive selection results in an elevated frequency of haplotypes carrying the advantageous allele at the expense of the others. Owing to the generations of recombination, long haplotypes are also rare. However, selection sweep creates haplotypes that are both long and frequent in a population (red and light red: right panel, middle and bottom). These methods are used to identify relatively recent and incomplete sweeps. (VIII) Elevated admixture contribution from one population: identifying sections of the genome with unusually high or low ancestry in a mixed population using MALD. Similar to VII, when two populations meet, one may carry a beneficial allele that can be later detected as a regional increase in ancestry, using a genome-wide map of highly differentiating population markers, and evaluated against the genome-wide expectation. I–VIII: blue line, genome-wide average.

**Figure 2.**
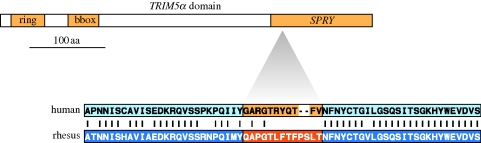
Increased number of function-altering mutations indicates a positively selected domain in *TRIM5*α** protein that mediates retroviral restriction (signature II). The tight clustering of humans versus rhesus non-synonymous changes in *TRIM5α* gene indicates a *SPRY* domain subjected to positive selection with an average d*N*/d*S* ratio of greater than four (Sawyer *et al*. 2005).

**Figure 3.**
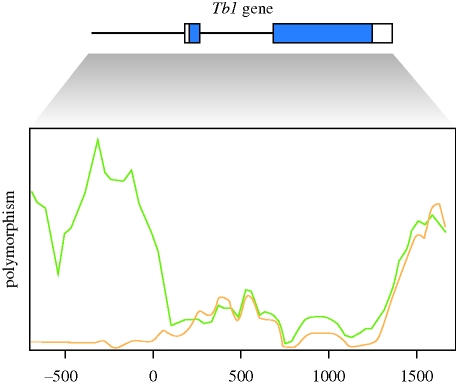
Reduced diversity to divergence ratio around the selected 5′ NTR variant of *Tb1* gene found in maize that causes the plant to carry ears instead of tassels (signature III). In the process of domestication, the 5′ NTR lost its variation, compared with the wild teosinte and the domesticated maize (Wang *et al*. 1999). Consistent with the selection hypothesis, the sliding window shows low polymorphism, but a high diversity in the region, evaluated as a signature of positive selection by the HKA test (Hudson *et al*. 1987). Yellow lines, maize; green lines, teosinte.

**Figure 4.**
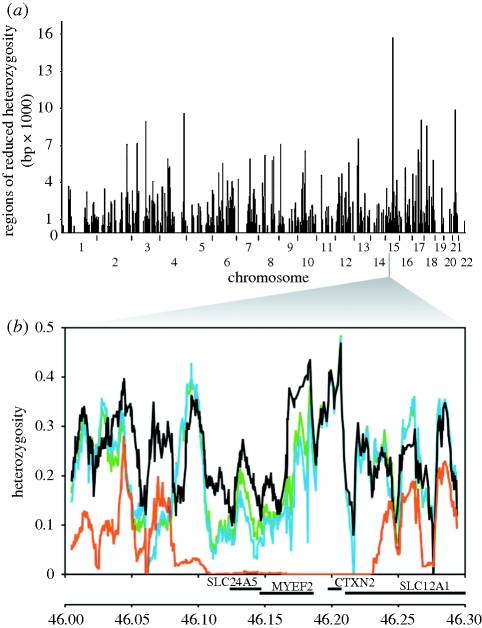
Reduced polymorphism around the *SLC24A5* gene involved in skin pigmentation indicates an episode of selection in the European population (signature IV). A region of decreased heterozygosity in Europeans (CEU) compared with Nigerian Yoruba (YRI), Chinese (CHB) and Japanese (JPT) people on chromosome 15 near the *SLC24A5* gene is significant when (*a*) compared across the genome in CEU samples and (*b*) plotted as averages in 10 kb intervals in the 300 kb vicinity of the gene, with heterozygosity for four HapMap populations (Lamason *et al*. 2005). Black lines, YRI; green lines, CHB; blue lines, JPT; orange lines, CEU.

**Figure 5.**
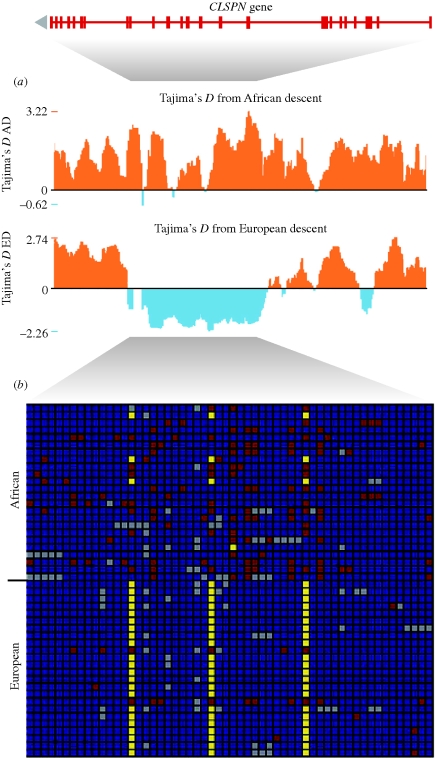
Example of a skewed frequency spectrum in the human *CLSPN* gene region indicating a positive selection signature in Europeans but not in Africans (signature V). A shift in frequency spectrum in the recently selected region is caused by the emergence of new low-frequency mutations. (*a*) Tajima's *D* values plotted across the *CLSPN* CRTR from the UCSC genome browser shows a region of negative values consistent with the sweep seen in (*b*), the visual genotype in the ED population adopted from Carlson *et al*. (2005). Each row corresponds to an individual, and each column corresponds to a polymorphic site in a visual genotype for 1.5 Mbp spanning the *CLSPN* CRTR in the Perlegen data. Common allele homozygotes are shown in blue, heterozygotes are shown in red, rare allele homozygotes are shown in yellow and missing data are shown in grey. The top 24 samples are African (AD); the bottom 23 samples are of European descent (ED). ED samples show much less variation, most of which comes as singleton mutations.

**Figure 6.**
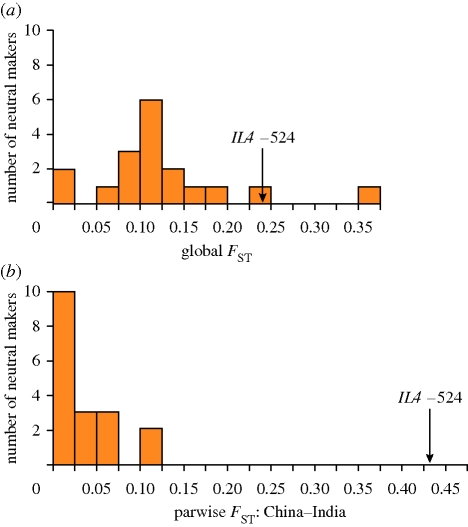
High population differentiation in *IL4*, a cytokine involved in immunity, may be attributed to positive selection (signature VI): a non-neutral pattern of differentiation at the *IL4* gene is demonstrated by evaluating the *F*_ST_ value at the *IL4* −524 locus against the same measure in a set of neutral loci elsewhere in the genome: (*a*) *F*_ST_ at −524 is higher, compared with 17 out of 18 neutral markers in a global distribution. (*b*) Pairwise *F*_*ST*_ at −524 between loci from China and India populations is dramatically elevated (adapted from Rockman *et al*. 2003).

**Figure 7.**
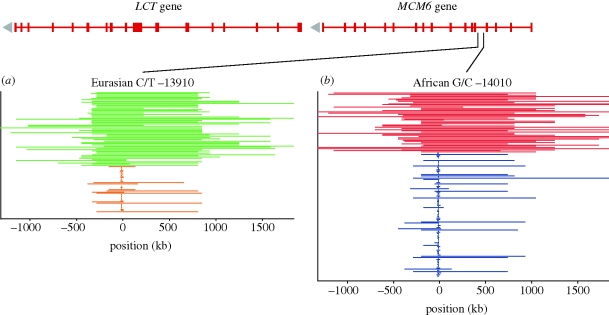
Unusual pattern of LD surrounding alleles indicates recent independent adaptations for post-adolescence lactase persistence: (*a*) *LCT*-C –14010 in Africans (red) and (*b*) *LCT*-T –13910 (green) in Eurasians (signature VII). Haplotypes, shown for each individual as parallel lines, are extended around the recently selected alleles, while the alternative alleles are enclosed by relatively short LD segments. In this example, haplotypes that surround lactase persistence (red and green) in Eurasians are much longer than the haplotypes that contain the alternative alleles (blue and orange). While the lactase-persistence alleles are different in the two populations, both are found in high frequencies and located on unusually long haplotypes (Tishkoff *et al*. 2007).

**Figure 8.**
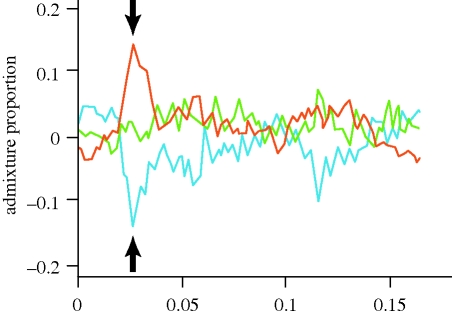
An excess of African and deficiency of European ancestry, as identified by admixture mapping (MALD) in Puerto Ricans, is evident in the region encompassing the HLA superlocus that contains diverse antigens essential in human immune function (signature VIII). Deviation in admixture proportion from three founder populations (African, European and Amerindian are represented by red, green and blue curves, respectively) is plotted along the physical location on chromosome 6 of Puerto Ricans. The *y*-axis indicates the excess/deficiency in ancestry at the corresponding SNP, averaged for 192 individuals (Tang *et al*. 2007). Orange lines, African; green lines, Native American; blue lines, European.

Traditionally, most tests for selection have concentrated on comparing a specific set of variable markers within a gene region against neutral expectations, empirically or from computer simulations. Recently, selection methods have been applied to newly available genome-wide SNP datasets. Genome-wide scans for evidence of historic selection events use either resequencing data from one or more species (Bustamante *et al*. 2005), or large collections of SNP polymorphisms from populations, e.g. the human HapMap populations (Altshuler *et al*. 2005; Frazer *et al*. 2007), to search for statistical departure from population genetic equilibrium (neutral) expectations as an indicator of a selected chromosomal region (Oleksyk *et al*. 2008). We list eight recently applied approaches to detect selection in genome-wide selection scans (GWSSs) in table 1 and illustrate them with examples in figures 1–8.

**Table 1.** General approaches and timing of detecting selection in genome-wide selection studies.

number	approaches	signatures	scope of the comparison	selection detected	time frame (years)^a^
*comparative, species-based*					
I	divergence rate and phylogenetic shadowing	reduction in the interspecific sequence divergence around a selected region relative to divergence of homologous regions genome-wide (Mayor *et al*. 2000; Ovcharenko *et al*. 2004) or when compared with a third species (Tajima 1993)	between species	positive, purifying	greater than 1 000 000
II	increased function-altering mutation rates	elevated ratio of non-synonymous (*N*) to synonymous (*S*) changes (d*N/*d*S*) in coding regions of selected genes compared with other genes evolving under the assumed neutrality (Nielsen & Yang 1998; Yang & Nielsen 1998)	within a species	positive	greater than 1 000 000
III	interspecies divergence versus intraspecies polymorphism	reduction in the ratio of intraspecific diversity to interspecific divergence (Hudson *et al*. 1987; McDonald & Kreitman 1991)	between species	positive	greater than 1 000 000
*population-based*					
IV	local reduction in genetic variation	a significant decrease in genetic variation (often measured as heterozygosity) around the selected site relative to its chromosomal neighbourhood or genome-wide (Oleksyk *et al*. 2008)	within a population	positive	less than 200 000
V	changes in the shape of the frequency distribution (spectrum) of genetic variation	a relative increase in the proportion of either low- or high-frequency mutations in the selected region (Tajima 1989; Fu & Li 1993; Fay & Wu 2000)	within a population	positive, balancing	less than 200 000
VI	differentiating between populations (*F*_ST_)	an increase or decrease in population differentiation in genomic regions under selection relative to the rest of the genome (Beaumont & Nichols 1996; Akey *et al*. 2002; Beaumont & Balding 2004)	between populations	positive, balancing	less than 80 000
VII	extended LD segments	extended LD producing remarkably long haplotypes around the beneficial SNP (Tishkoff *et al*. 2001; Sabeti *et al*. 2002; Voight *et al*. 2006)	within a population	positive	less than 30 000
VIII	elevated admixture contribution from one population (MALD)	detecting a relative excess or decrease in admixture contribution within a selected region by one of the populations (Tang *et al*. 2007)	in a population after admixture	positive	less than 500

^a^The times for I–II are based on the date of human–chimpanzee divergence (5 Myr ago). Time estimates for III–VII are from Sabeti *et al*. (2005). Time estimates for VII are based on the assumptions outlined in Smith & O'Brien (2005). All estimates are for the human lineage.

Computational analytical approaches to genome-wide scans for selection can be divided into methods using sequence divergence and diversity patterns between species and methods that consider genetic variation from populations (table 1). Generally, between-species comparisons are used to identify older events, while population-based methods reveal more recent episodes of selection (table 1). Discovery of the same selected gene regions using alternative approaches can provide cogent evidence for selective influences in the region. However, the success of one test and the failure of a second does not preclude selection in a genomic region because different methods will track different intervals of a population's history (Sabeti *et al*. 2006; Kelley & Swanson 2008) (table 1).

In this review, we describe eight distinctive signatures of selection that capture different evolutionary mechanisms and relative time scales (table 1). We then describe good examples of genes where selection has been demonstrated. Finally, we compare various approaches from different GWSSs applied to human genome-wide datasets and assess independent replication of putative regions found by different methods and study populations.

## 2. Detecting selective sweeps using between-species comparisons

### (a) Divergence rate and phylogenetic shadowing

In contrast to the demographic processes acting upon the entire ensemble of genomic diversity, natural selection targets primarily functional elements in specific gene regions. While mutation and recombination restore variation in the adjacent sites, selected non-synonymous changes persist in the genome, changing the overall pattern of divergence and/or diversity. Selection signatures can be observed by plotting the between-species divergence of homologous segments and comparing it with the genome-wide average: phylogenetic shadowing (Mayor *et al*. 2000; Ovcharenko *et al*. 2004). The less-variable segments can be interpreted as either purifying selection, or past actions of positive selection. Divergence rates can also be evaluated by comparing homologous sequences using a third species as an outgroup (Tajima 1993).

Phylogenetic shadowing quantifies the amount of divergence among homologous sequences between two or more species (Mayor *et al*. 2000). Using parsimony, the rate of substitution can be considered on a phylogenetic tree (Blanchette *et al*. 2002). Regions affected by purifying selection are significantly less divergent than the genome-wide means. Phylogenetic shadowing has been particularly useful in identifying putative regulatory elements in non-coding DNA (Blanchette *et al*. 2002). The advantage of phylogenetic shadowing is that it takes into consideration the underlying evolutionary context, although assessment is difficult when confident alignment of regions between species decays.

Predictions for positive selection detected by looking at the relative rates of divergence between homologous species are not clear at this time, and more effort is needed to develop appropriate statistical approaches to formally incorporate phylogenetic shadowing for identifying different types of selection. However, these methods can detect parts of a genome sequence being conserved by the action of purifying selection among different species (Zhang & Gerstein 2003), and this approach has been incorporated into computational algorithms (Mayor *et al*. 2000).

### (b) Increased function-altering mutation rates

The rates at which non-synonymous mutations are retained in a population indicate the presence and strength of selection in a coding gene. An unusually high number of function-altering (non-synonymous) changes from a comparison between two homologous sequences can point to the genomic regions where past episodes of positive selection may have taken place (figure 2). The rate of mutation is expressed as the number of substitutions per non-synonymous site (d*N* or *Ka*) or the number of substitutions per synonymous site (d*S* or *Ks*). In neutrally evolving sequences, no difference should be observed between the two measures, or d*N* = d*S*. Positive selection in a region results in an increase in the number of non-synonymous mutations, such as d*N* > d*S* (or *Ka* > *Ks*) (see example in figure 2). Conversely, if functional mutations are constantly removed from a population by purifying selection, the opposite trend can be expected: d*N* < d*S* (or *Ka* < *Ks*). The ratio (**ω** = d*N*/d*S*) is evaluated among different coding regions.

d*N/*d*S* tests have been used extensively. Typically, they contrast likelihood ratio of data under the null hypothesis, assuming neutrality to various alternative hypotheses. A twofold difference between the log likelihoods follows a *χ*^2^ distribution, and if the value is found in a critical region, neutrality can be rejected and selection is inferred (Nielsen & Yang 1998; Yang & Nielsen 1998).

### (c) Interspecies divergence versus intraspecies polymorphism

Under the assumption of selective neutrality, the proportion of synonymous (d*S*) and non-synonymous (d*N*) changes should be the same for polymorphism within the species as for divergence between species (figure 1*a*). Conversely, purifying selection removes non-synonymous mutations faster, causing a lower d*N* value between, rather than within species. Two main tests that compare d*N* and d*S* between and within species have been used to detect selected regions: (i) the McDonald–Kreitman (MK) test that contrasts synonymous and non-synonymous sites of a gene segment within and between species (McDonald & Kreitman 1991) and (ii) the Hudson–Kreitman–Aguade (HKA) test that contrasts polymorphism and divergence among multiple loci (Hudson *et al*. 1987). The latter is an extension of the former and is based on the assumption that under neutrality, polymorphism and divergence are the same for all neutrally evolving genes. Therefore, a candidate gene compared with one or multiple putatively neutral loci, and the deviation in the ratio of polymorphism to divergence can be evaluated. A low ratio of intraspecies diversity versus between-species divergence in and around a candidate gene can be interpreted as signature of positive selection (see examples in figures 1(III), 2 and 3), whereas a decreased divergence could be interpreted as the action of purifying selection.

Between-species genomics tests (I–III) can be used to identify very old selections (table 1); however, they require many site changes to exceed the background of mutational drift over long intervals of species differentiation and have limited ability to narrow the time when selection occurred. In addition, they cannot precisely identify a single selected site allele. By contrast, studies based on the population data can be used to detect recent selection, to estimate the time interval of selection events and, in some cases, to identify selection acting on a single nucleotide.

## 3. Detecting selective sweeps from population data

### (a) Local reduction in genetic variation

An important genomic indicator of a selective sweep involves local reduction in variation within a selected gene and in adjacent SNP variants (Maynard Smith & Haigh 1974) (see example in figure 4). Local reduction in genetic diversity can persist for a long time, and indicate selection across a long genomic region; i.e. if an allele with a selective advantage of one per cent will generate a homozygous region of an estimated 600 kb (Mikkelsen *et al*. 2005), this selection makes finding an actual selected gene more difficult.

While scans for diminished polymorphism are easily implemented, several caveats can influence their interpretation. First, this signature may be difficult to distinguish from the effects of demographic history because population bottlenecks or recent founder effects can reduce polymorphism across the genome of derivative populations. SNP analyses of domestic dogs and cats both show long stretches of alternating heterozygous and homozygous regions as a consequence of domestication and breed development, masking any gene-based selection in their recent past (Lindblad-Toh *et al*. 2005; Pontius *et al*. 2007). However, in most outbred species, a selected region would display local SNP homozygosity, compared with abundant polymorphism elsewhere in the genome (Oleksyk *et al*. 2008).

### (b) Changes in the shape of the frequency distribution *(*spectrum*)* of genetic variation

After a selective sweep reduces variability around a selected site, new mutations will gradually appear. These mutations would initially occur at low frequencies because their chances of increasing in a population under neutral drift are very low, and it takes some time after the sweep to restore a more typical distribution of mutation frequencies in a region (a frequency spectrum) that is consistent with the action of neutral forces. This shift to a low-frequency spectrum of polymorphism constitutes a signature of positive selection (Tajima 1989). Alternatively, balancing selection maintains a high proportion of the high-frequency polymorphisms, thereby shifting the spectrum to the intermediate frequencies.

A shift in frequency spectrum is used in selection tests in one of two distinct ways: (i) changes in the spectrum (i.e. clustering of rare alleles in a region) and (ii) changes in the occurrence of ancestral and derived alleles. The former approach is captured by Tajima's *D* test, which compares the mean pair-wise difference between sequences in a population sample (**π**) with the number of differences estimated using the number of polymorphic sites (*s*) (figure 5). Tajima's *D* equals zero for neutral variation, is positive when an excess of rare polymorphism indicates positive selection and is negative in the excess of high-frequency variants, indicating balancing selection (Tajima 1989). The second approach exploits the fact that polymorphism within the selective sweep leaves excess derived alleles that hitchhike on selected haplotypes. Derived alleles arise by mutation, and are expected to have lower allele frequencies than their ancestral counterparts because of their relatively younger age. A selective sweep creates a situation where too many derived alleles are found at high frequencies. There are several examples of tests using the derived allele approach. For example, Fu and Li's *F* test counts the number of derived alleles observed only once and compares it with the average pair-wise difference between species (Fu & Li 1993), while Fay and Wu's *H* test compares the number of derived alleles either at low or high frequencies with the number of variants at the intermediate frequencies (Fay & Wu 2000).

Tests based on the frequency spectrum of rare or derived mutations have been implemented in studies of human and non-human species (Hughes & Yeager 1998; Seltsam *et al*. 2003; Bersaglieri *et al*. 2004; Stajich & Hahn 2005; Civetta *et al*. 2006; de Meaux *et al*. 2008; Ojeda *et al*. 2008). The next challenge is to apply them to genome-wide data. However, as available SNP datasets were obtained by genotyping previously discovered variants, an ascertainment bias for enrichment of high-frequency polymorphisms and paucity of low-frequency variants arises, biasing the performance of these tests (Nielsen *et al*. 2005). Attempts to rectify this situation have been made by incorporating information from the genotyping protocols into selection tests (Nielsen & Signorovitch 2003; Nielsen *et al*. 2005). In addition, some human genomic datasets such as HapMap are being expanded with an effort to control for the ascertainment (Frazer *et al*. 2007). Unfortunately, for non-human species, relief from an ascertainment bias will not soon be readily available, and genome-wide scans for selection using the frequency spectrum will continue to suffer from this problem until reliable and inexpensive data from the next-generation whole genome sequencers become available.

Demographic processes change genome-wide patterns of genetic variation by altering effective population size independently of natural selection. Various demographic events can interfere with the selection signal detected by these methods. Population expansion could increase the proportion of low-frequency variants, mimicking the effect of selection sweep identified by the spectrum methods described in §3*b* (Nielsen *et al*. 2005). A population bottleneck could produce an excess of intermediate frequency variants, resulting in a spectrum close to that produced by balancing selection.

Tests based on derived allele frequencies seem to be less sensitive to the demographic events than those based either on a reduced amount of polymorphism or on finding a shift in the rare/common allele frequency. Yet, these signatures seem to be relatively short-lived as derived alleles are lost, and also suffer from population subdivision (Przeworski 2002). Identification of derived alleles requires phylogenetic knowledge of the ancestral states that are determined by aligning sequences between closely related species. In humans, determination of ancestral states is currently facilitated by the availability of whole genome sequence from great apes. Soon, the ancestral state will be inferred by comparison with the Neanderthal genome or even genomes of other human populations, given the improved knowledge of human population history. However, for non-human species, the ancestral allele information may not be so easily available until related genome sequences become available.

### (c) Differentiating between populations *(F*_ST_*)*

Variation of local conditions imposes differential selection pressures shaping variable adaptive landscapes (Wright 1951). Recent adaptations in populations often reflect the peculiarities of local environments. Local conditions are different from one locality to another and differ considerably between ecosystems. In some instances, given enough geographical isolation restricting gene flow, selection signatures could differ considerably between populations. Consequently, regions experiencing selective sweeps, in addition to the decreased variation within the population, should also display increased levels of population differentiation, a measure commonly denoted as *F*_ST_ (Wright 1951).

Tests that look for population differentiation are based on the premise that natural selection can change the amount of differentiation between different populations of a species. Unless a selective sweep has already spread to all populations, the amount of genetic differentiation within the region that includes selected locus will increase. Therefore, if genetic differentiation in the genomic region is greater than the level expected under neutrality, this differentiation may be a consequence of natural selection (see example in figure 6).

The Lewontin–Krakauer test represented the earliest effort to incorporate interpopulation differences: it compared the level of genetic differentiation among populations with that predicted by a specific neutral model using a standard variance ratio test (Lewontin & Krakauer 1973, 1975). This approach was criticized as unreliable (Nei & Maruyama 1975), but in the past decade it has been revisited several times. One approach generated a distribution of *F*_ST_ under a neutral model of population structure to build an expected distribution conditioned on the initial allele frequencies. Outliers identified by comparing observed values with this conditioned distribution exhibit signatures of selection (Bowcock *et al*. 1991). This approach has been extended to use a coalescent model to generate an expected distribution of *F*_ST_ conditional on heterozygosity (Beaumont & Nichols 1996), and to use a Bayesian model implemented through Markov Chain Monte Carlo simulations (Beaumont & Balding 2004). Alternatively, some studies rely on sampling a large number of loci across the genome: these resampling-based tests compare the levels of genetic differentiation of one or more loci with the genome-wide (or chromosome-wide) distribution of *F*_ST_ (Akey *et al*. 2002; Oleksyk *et al*. 2008). The outliers found in this manner can be compared with the outliers found by other approaches (table 1). Those regions showing both signatures are more likely to harbour multiple selection signatures than those showing only the increased levels of *F*_ST_ (Oleksyk *et al*. 2008).

Considerable differences in the *F*_ST_ values around the selected site could be affected by polymorphism frequency at the onset of positive selection. For instance, those variants present on the beneficial haplotype displaying high heterozygosity values would accumulate little differentiation between a population selected for that haplotype and a population lacking the selection pressure. Those selected variants initially at low frequencies could lead to large differences between populations, under the condition that the chromosomal region initially has enough variation in the flanking sites, so the resulting differentiation could be detected.

Differentiation among the populations is also sensitive to demographic factors, including both migration and genetic drift. To avoid this problem, recent scans started to take advantage of large-population datasets, and compare outlier loci with the empirical distribution of population differentiation across the genomes of compared populations (Oleksyk *et al*. 2008). Alternatively, some scans use computer simulations employing realistic demographic conditions to obtain values of population differentiation expected under the assumption of neutrality (Beaumont & Balding 2004).

### (d) Extended linkage disequilibrium segments

Historic selective sweeps in population data are apparent because of a hitchhiking effect described by Maynard Smith & Haigh (1974). As selection acts not on genotypes but on individuals carrying adaptive phenotypes that gain reproductive advantage, beneficial mutations, along with the entire genomes, are selected. However, independent assortment and recombination reshuffle chromosomes and regions distal to a selected beneficial variant.

A selective sweep region would contain many neutral variants tightly linked to the beneficial mutation on haplotypes limited in length by a combination of selection strength and recombination rate. The extent of this association depends on the recombination distance, so persistence of a frequent, unusually long haplotype indicates strong, recent or ongoing selection, especially if that haplotype has risen to high frequency. Over many generations, haplotype size becomes smaller owing to recombination with other haplotypes (see example in figure 7).

Extended linkage disequilibrium (LD) tests are useful for detecting partial selective sweeps, with allele frequencies as low as approximately 10 per cent (Sabeti *et al*. 2002; Voight *et al*. 2006), and they are relatively robust to the choice of genetic markers used or ascertainment bias (Sabeti *et al*. 2002). An unusual LD pattern is detected in three selection tests. First, the extent of haplotype diversity (SNP variant within a haplotype-defined region) can be assessed by comparing the diversity of haplotypes carrying the selected variant with all the allelic haplotypes that carry the other SNP alleles. Haplotypes carrying a selected allele are expected to display lower diversity as they all originate from a subset of chromosomes carrying the beneficial variant (Tishkoff *et al*. 2001). Second, the extended haplotype homozygosity (EHH) test evaluates length and frequency of haplotypes in a population (Sabeti *et al*. 2002). As it takes a long time to reach high frequency by genetic drift alone, the frequent older haplotypes experience more recombination, and decrease in length. In contrast, younger alleles tend to be longer, but at lower frequencies. Alleles that have both high-frequency and long-range LD with other alleles (long-range haplotype homozygosity) are evidence for a selective sweep. The relative extended haplotype homozygosity (REHH) test computes EHH of a single haplotype to the EHH of allelic haplotypes in the same genomic region (Sabeti *et al*. 2002). Third, the integrated haplotype score (iHs) test compares the EHH decay around ancestral and derived alleles (Voight *et al*. 2006).

LD extension tests are the most useful for the identification of recent, incomplete sweeps (Sabeti *et al*. 2006), but they require genetic phase data to define the haplotypes explicitly. In addition, to be robust, LD-based GWSSs would require precise control for regional variation in the recombination rate, as ‘cold spots’ for recombination not under selection can mimic extended haplotypes. After 30 000 years, a typical human chromosome will have undergone more than one crossover per 100 kb (Sabeti *et al*. 2002). The remaining short fragments may be too short to detect selection by an LD test.

### (e) Excess or decrease in admixture contribution from one population mapping by admixture linkage disequilibrium

Admixture mapping, also called mapping by admixture linkage disequilibrium (MALD) is a novel method that aims to localize disease-causing genetic variants that differ in frequency across populations (Smith & O'Brien 2005). It is most useful in admixed populations such as in African-Americans (Smith *et al*. 2004), Latinos (Price *et al*. 2007) and Puerto Ricans (Choudhry *et al*. 2008), i.e. modern populations that descended from a recent mix of ancestral groups that had been geographically isolated for long evolutionary time. The approach considers that a genomic region of a disease-causing gene would show a higher percentage of detectable genomic ancestry from the parent population that has greater risk for the disease (Chakraborty & Weiss 1988; Briscoe *et al*. 1994; Smith & O'Brien 2005). For example, Puerto Ricans carry an excess of African admixture in an HLA region of chromosome 6, an excess of Native American admixture in two other regions (on chromosomes 8 and 11) and a corresponding deficiency in European admixture at the same genomic locations, suggesting an historic adaptive advantage for these regions during admixture (Tang *et al*. 2007) (figure 8). While there has been a discussion whether or not the long range LD can potentially confound signals of selection in admixtured populations like the one used in this study (Price *et al*. 2008; Tang *et al*. 2008), it remains to be seen whether such recent selection signatures can be found in other admixed populations.

## 4. Examples of selected regions discerned from candidate gene studies

Table 2 lists 30 examples of genes under selection based upon various approaches reviewed above. We discuss five of these selected genes (*LCT*, *MC1R*, *CCR5*, *FY* and *G6PD*) in detail because they have been well represented in the literature and give a good representation of evidence, mechanisms and evolutionary time scale for instances of human selection.

**Table 2.** Candidate genes, the tests used to identify selection and GWSSs that found them. (The candidate genes with any evidence of selection found by genome scan are in bold. n.a., not applicable.)

chromo- some	gene	location	author	discovered by	test(s)	population(s)	found by scan (same locus)	found by scan (nearby locus)
1	***FY***	1q21–q22	Hamblin & Di Rienzo (2000)	frequency spectrum, population differentiation	Fay and Wu's H, *F*_ST_	Africans		by 50 kb (Frazer *et al*. 2007)
1	*AGT*	1q42–q43	Nakajima *et al*. (2004)	unusual LD	tight LD	Africans		
1	*ASPM*	1q31	Mekel-Bobrov *et al*. (2005)	comparative methods	*Ka/Ks*	World		
2	***LCT***	2q21	Bersaglieri *et al*. (2004)	unusual LD	iHs, EHH	Europeans, World	Frazer *et al*. (2007); Nielsen *et al*. (2005); Voight *et al*. (2006)	
2	*CAPN10*	2q37.3	Fullerton *et al*. (2002)	population differences	*F*_ST_	Africans versus non-African		
3	***CCR5***	3p21.31	Stephens *et al*. (1998)	population differences	*F*_ST_ and low heterozygosity	Europeans	Oleksyk *et al*. (2008)	
4	***ADH1B***	4q21–q23	Osier *et al*. (2004, 2002)	unusual LD, and population differences	LD, *F*_ST_	Asians	Frazer *et al*. (2007)	by 100 kb (Voight *et al*. 2006)
5	***IL13***	5q31	Sakagami *et al*. (2004)	population differences	*F*_ST_	World		by 200 kb (Oleksyk *et al*. 2008)
5	***IL4***	5q31.1	Rockman *et al*. (2003)	population differences	*F*_ST_	World		by 200 kb (Oleksyk *et al*. 2008)
6	***HFE***	6p21.3	Toomajian & Kreitman (2002)	multiple	*Ka/Ks*, LD, *F*_ST_	Asians		by 100 kb (Voight *et al*. 2006)
6	***TRPV6***	7q33–q34	Akey *et al*. (2006)	low diversity and frequency spectrum	Tajima's *D*, and low diversity	Africans		at different coordinates (Carlson *et al*. 2005)
7	***CYP3A5***	7q21.1	Thompson *et al*. (2004, 2006)	frequency spectrum	Tajima's *D*	Europeans, Asians	Carlson *et al*. (2005); Oleksyk *et al*. (2008)	by 200 kb (Voight *et al*. 2006)
7	***FOXP2***	7q31	Enard *et al*. (2002)	comparative methods	*Ka/Ks*	World	Oleksyk *et al*. (2008)	
8	*MCPH1*	8p23.1	Evans *et al*. (2006*a*)	comparative methods	*Ka/Ks*	World	Bustamante *et al*. (2005) (negative)	
8	*NAT2*	8p22	Patin *et al*. (2006)	unusual LD	REHH	Europeans		
9	*CDK5RAP2*	9q33.2	Evans *et al*. (2006*b*)	comparative methods	*Ka/Ks*	World		
10	*FGFR2*	10q26	Goriely *et al*. (2003); Goriely *et al*. (2005)	n.a.	distribution of mutations in sperm skewed	World		
11	*DRD4*	11p15.5	Ding *et al*. (2002); Wang *et al*. (2004)	unusual LD	LD	World		
11	***HBB***	11p15.5	Ayodo *et al*. (2007)	population differences	*F*_ST_	Africans		by 100 kb (Frazer *et al*. 2007)
11	*MMP3*	11q22.3	Rockman *et al*. (2004)	low diversity and population differences	*F*_ST_, low heterozygosity,	Europeans		
11	*CASP12P1*	11q22.3	Xue *et al*. (2006)	frequency spectrum and unusual LD	Tajima's *D*, Fay and Wu's H, LD	World		
13	***CENPJ***	13q12.12	Evans *et al*. (2006*b*)	comparative methods	*Ka*/*Ks*	World	Frazer *et al*. (2007)	by 200 kb (Voight *et al*. 2006)
15	***SLC24A5***	15q21.1	Lamason *et al*. (2005)	low diversity	Low heterozygosity	Europeans	Sabeti *et al*. (2007)	by 80 kb (Voight *et al*. 2006)
16	*MC1R*	16q24.3	Makova & Norton (2005); Makova *et al*. (2001)	low diversity and frequency spectrum	pi, theta, Fu and Li F	Africans, Europeans, Asians		
17	***TTL.6***	17q21.32	Chen *et al*. 2006)	comparative methods	*Ka*/*Ks*	World		by 50 kb (Akey *et al*. 2002)
X	***DMD***	Xp21.2	Nachman & Crowell (2000)	reduced variation and unusual LD	HKA, LD	non-African	Frazer *et al*. (2007)	
X	*CD40LG (TNFSF5)*	X26	Sabeti *et al*. (2002)	unusual LD	EHH	Africans		
X	*FIX*	Xq27.1–q27.2	Harris & Hey (2001)	low diversity and frequency spectrum	low diversity, Tajima's *D*	World		
X	*G6PD*	Xq28	Sabeti *et al*. (2002); Tishkoff *et al*. (2001)	unusual LD	EHH	Africans		
X	*MAOA*	Xp11.3	Andres *et al*. (2004); Gilad *et al*. (2002)	comparative methods	*Ka*/*Ks*	World		

### (a) Lactase (*LCT*) gene and post-adolescence lactase expression persistence

The lactase enzyme is encoded by a single gene (Boll *et al*. 1991) on chromosome 2q21 (Harvey *et al*. 1993). In Europe, three common *LCT* haplotypes (A, B and C) were identified encompassing the gene. Haplotype A is the most common in northern Europe (86%) where lactase expression persistence after adolescence is common, but less common in Southern Europe, as well as in other world populations such as in India, Africa and Asia, where lactase expression persistence past adolescence is rare (Hollox *et al*. 2001).

It has been hypothesized that a derived T variant of the adjacent *MCM6* gene at position −13910 (A/T) in the A haplotype is responsible for lactase persistence in Eurasia (Enattah *et al*. 2002; Poulter *et al*. 2003). This *MCM6-*T variant is absent or extremely rare in most African populations (Mulcare *et al*. 2004). Several *in vitro* studies indicate that *MCM6* acts as a *cis*-regulatory element that upregulates a promoter region of the *LCT* gene (Olds & Sibley 2003; Troelsen *et al*. 2003; Lewinsky *et al*. 2005). However, it has been suggested that a different variant (C), located at −14010 (G/C), is responsible for lactase persistence in Africans (Tishkoff *et al*. 2007). If these inferences are affirmed, then lactose persistence evolved independently as a response to selective pressures in different parts of the world (figure 7).

Recent selection about the *LCT* locus is supported by several tests. There was an excess of high *F*_ST_ values for the 99 flanking DNA sites on either side of the *LCT* locus (Bersaglieri *et al*. 2004). Signatures of selection were present when interpopulation differentiation was corrected using *P*_excess_: a measure that reflects the rise in frequency of the flanking variants relative to their original value derived from its distribution in populations that did not experience selection at the same variant (Bersaglieri *et al*. 2004). This, in effect, is an equivalent to the reduction in local variation. Finally, REHH was estimated to be extremely high (13.2), indicating that the lactase-persistence haplotype displayed homozygosity over more than 800 kb, much longer than that displayed by the lactase non-persistent haplotypes (Bersaglieri *et al*. 2004). The −14010 C allele for lactase-persistence alleles was included in the analysis; it was also at a high frequency and found on a long haplotype in African populations (Tishkoff *et al*. 2007). Consequently, selection in the *LCT* locus is evidenced both by high population differentiation and a local decrease in genetic variation, and by the unusual pattern of LD. All three signatures of selection are consistent with the current hypothesis of the multiple origins of lactase persistence in the very recent (less than 7000 years) human evolutionary history, probably associated with the origins of human agricultural development (Enattah *et al*. 2005; Tishkoff *et al*. 2007).

### (b) Melanocyte receptor gene and skin colour

The melanocyte receptor (*MC1R*) gene is located at chromosomal position 16q24.3 in humans. A recent genome-wide association scan confirmed the role of *MC1R* SNPs in hair, eye and skin pigmentation (Sulem *et al*. 2007). This gene was thought to consist of a single exon until a possibility of alternative splicing was suggested (Tan *et al*. 1999). Consequently, the gene may have another exon at the 3′ end encoding 65 amino acids, but its function is unknown. *MC1R* is a switch that determines the relative proportion of pigment produced by a melanocyte. The active form of the gene produces eumelanin (dark pigment). The inactive form results in a prevalence of pheomelanin (light pigment). Thus, loss-of-function mutations at *MC1R* could result in a spectrum of pigment variation: from light brown to yellow (Robbins *et al*. 1993). *MC1R* is also associated with red hair phenotypes (Healy *et al*. 2001), and a characteristic of a homozygous *MC1R* null individual is red hair and fair skin (Beaumont *et al*. 2008). In non-human species, deletions in the *MC1R* gene are implicated in light and melanistic phenotypes in domestic and wild species (Barsh 1996; Marklund *et al*. 1996; Kijas *et al*. 1998; Newton *et al*. 2000; Eizirik *et al*. 2003).

While *MC1R* is a small gene, it is highly variant, often with phenotypic consequences (Garcia-Borron *et al*. 2005). Specific mutations also link *MC1R* to different forms of skin cancer, including melanoma (Smith *et al*. 1998; Kanetsky *et al*. 2006; Fernandez *et al*. 2007). *MC1R* coding SNPs in human populations in Africa are predominantly synonymous: eight synonymous versus three non-synonymous (Harding *et al*. 2000), and non-synonymous changes are absent outside of South Africa (John *et al*. 2003). By contrast, European polymorphisms are largely non-synonymous: two synonymous versus 10 non-synonymous (Harding *et al*. 2000). Recently, 20 more non-synonymous changes have been identified in Europeans (Makova & Norton 2005). Fewer *MC1R* variants occur in Africa, compared with non-African populations, which sharply contrasts with African populations showing greater genome-wide diversity than the non-African ethnicities (Gerstenblith *et al*. 2007).

Selection signatures around *MC1R* are complex. The d*N*/d*S* ratio for *MC1R* between humans and chimpanzees is unusually high (0.63), compared with the genomic background of approximately 0.25. The evolutionary transition may have evolved from light skin covered with hair (as in forest-dwelling chimpanzees) to dark skin in early humans (Rogers *et al*. 2004). Based on the pattern of variation at *MC1R*, most studies agree that natural selection in Africa is of a purifying nature (Rana *et al*. 1999; Harding *et al*. 2000). This may be explained by individuals with fair skin experiencing selective disadvantage in the African environment with its intense sunlight: fair-skinned individuals are at higher risk of several types of skin cancer (Rogers *et al*. 2004).

Outside of Africa, the *MC1R* gene experienced an adaptive differentiation: large *F*_ST_ values exist for the non-African populations, particularly between Asians and all other populations (Savage *et al*. 2008). Controversy exists as to whether the non-African populations experienced relaxation of the purifying selective constraint still acting in Africa (Harding *et al*. 2000), or whether those dark-skinned individuals living in high-latitude regions are at higher risk for diseases caused by deficient or insufficient vitamin D levels, resulting in the diversifying mode of selection (Rana *et al*. 1999; Parra 2007). The hypothesis of relaxed pressure on *MCM6* outside Africa is supported by the evidence based on MK and HKA tests (Harding *et al*. 2000; John *et al*. 2003). The alternative hypothesis of vitamin D deficiency in Europe has been supported by the evidence from the tests evaluating the frequency spectrum of mutations (Tajima's *D*) (Harding *et al*. 2000; Savage *et al*. 2008). The difference between the evolutionary time scale of these tests (greater than 200 000 to less than 200 000 years; table 1) may reflect a shift in alternate selection modes in Europe. Particularly, positive selection may operate in Southern Europeans, specifically in Greeks, Italians and Spanish, based on significant Tajima's *D* values (Savage *et al*. 2008). Finally, some degree of weak positive selection may even be present in northern European populations, possibly reflecting an adaptation to vitamin D deficiency (Sulem *et al*. 2007; Savage *et al*. 2008).

### (c) Duffy blood group *(*FY*)* gene and malaria

The *FY* gene (chromosome 1p21–q22) encodes the Duffy antigen chemokine receptor (DARC), which is expressed on the membrane of erythrocytes and other lymphoid tissues. While the normal physiological function of the DARC is unclear, the malarial parasite (*Plasmodium vivax*) requires DARC to gain entry into a cell (Livingstone 1984; Hadley & Peiper 1997). The resistance allele (FY*0) has been localized to a single nucleotide base substitution (T/C) of the ancestral allele (FY*B) at nucleotide −46 of the promoter region (Chaudhuri *et al*. 1995; Tournamille *et al*. 1995; Seixas *et al*. 2002). This change eliminates the receptor in erythrocytes only, while other cells carrying it remain unaffected (Hadley & Peiper 1997). Malaria resistance was suggested as an explanation for the elevated frequencies of the Duffy FY*0 allele in African populations. As the highest frequencies of FY*0 are found in the regions where *P. vivax* is either completely absent or present at low frequencies, Livingstone (1984) suggested further that a different agent may have increased FY*0 frequencies some time before malaria, creating a pre-adaptation that prevented *P. vivax* from becoming endemic in those areas. *Plasmodium vivax* is closely related to Asian primate malaria vectors, and Mu *et al*. (2005) have speculated that the pathogen may have emerged from *Macaca* to humans 53 000–265 000 years ago, and entered Africa afterwards.

Available data for the *FY*-Duffy locus situation presents a compelling case for a gene affected by selection owing to the extreme differentiation between populations (*F*_ST_) from different continents (Lautenberger *et al*. 2000). Recent evidence shows that *F*_ST_ values are the greatest for the polymorphic sites nearest to the presumed selected variant, but diminish in the flanking regions (Hamblin *et al*. 2002). However, detecting additional selection evidence has not been straightforward. For example, the Duffy region shows a skew towards rare variants in African populations, indicating a possibility of positive selection, but the Tajima's *D* values have not been significant (Hamblin *et al*. 2002). Compared with the European population, Africans display a two- to threefold decrease in genetic variation, including the upstream region (Hamblin & Di Rienzo 2000). In addition, positive selection was supported by the HKA tests comparing polymorphism at the *FY* locus with presumably neutral and unlinked loci (Hamblin & Di Rienzo 2000). Finally, there is evidence of positive selection in the excess of the high-frequency-derived variants measured by Fay and Wu's tests (Fay & Wu 2000; Hamblin *et al*. 2002). The time frame for selection at *FY* has been estimated to 6500–97 000 years (Hamblin & Di Rienzo 2000). This is both consistent with the time frame of selection approaches involved (table 1, III–VI) and overlaps with the date for the switch of the malaria parasite from a primate to a human host (Mu *et al*. 2005).

### (d) Glucose-6-phosphate dehydrogenase (*G6PD*) gene and malaria

The *G6PD* gene is located at the telomeric region of the X chromosome localized to q28, and it consists of 13 exons spanning 18 kb. Mutants showing 100 per cent deficiency in the G6PD enzyme have gross deletions, nonsense or frame-shifting mutations that are incompatible with life (Beutler 1994). Chimpanzees have several amino-acid variants, and the overall variation pattern at *G6PD* in primates in general can be explained by recent purifying selection as well as by a strong functional constraint dating back to at least tens of millions of years. In that context, the recent signature of positive selection at *G6PD* in humans is interesting (Verrelli *et al*. 2006).

The endemic spread of malaria, especially the variety caused by *Plasmodium falciparum*, generally associated with the spread of agriculture 10 000 years ago, is generally regarded as one of the strongest known selective pressures in the recent human evolution. *Plasmodium falciparum* breaks down haemoglobin, and this process releases potentially toxic by-products, including iron, which is a source of oxidative stress. Deficiency in G6PD, a pivotal enzyme in the pentose phosphate metabolic pathway that protects against oxidative stress, simultaneously increases the resistance to malaria (Kwiatkowski 2005). Not surprisingly, geographical distribution of G6PD deficiency has been shown to be consistent with the action of selection for malarial resistance (Ganczakowski *et al*. 1995).

The overall level of nucleotide heterozygosity at *G6PD* is typical of other genes on the X chromosome, compatible with the neutral expectation (Saunders *et al*. 2002). However, selection has affected genetic variability over long distances along the flanking chromosome, creating an extended LD around the protective mutation detected by EHH (Sabeti *et al*. 2002). Selection evidence for *G6PD* is generally consistent with the hypothesis of recent positive selection. One of the haplotypes (A-allele) arose within the past 3840–11 760 years, and the other (Med allele) arose within the past 1600–6640 years (Tishkoff *et al*. 2001).

### (e) Chemokine receptor 5 (*CCR5*) gene and infectious diseases

The chemokine receptor 5 (*CCR5*) gene is localized on chromosome 3p21 and contains four exons but only two introns, spanning approximately 6 kb. The gene is expressed predominantly in T cells, dendritic cells, microglia and macrophages and is likely to be involved in the inflammatory responses to infection (O'Brien & Nelson 2004). The most notable polymorphism in the *CCR5*-*Δ32* blocks HIV-1 infection (Dean *et al*. 1996; Carrington *et al*. 1999), but HIV-1 susceptibility and time to progression to AIDS have been associated with other *CCR5* polymorphisms, many of them located in the 5′ *cis*-regulatory region of the gene (Carrington *et al*. 1997; Mummidi *et al*. 1997; Martin *et al*. 1998).

While HIV has emerged on the global scale only recently, population genetic data strongly suggest that *Δ*32 has been under selection pressure for a long time (Stephens *et al*. 1998; Bamshad *et al*. 2002; Novembre *et al*. 2005). The *Δ*32 variant is highly localized in the northern European population, where frequencies are as high as 16 per cent in Scandinavian populations, and gradually decreases across Eurasia; results are very high, with *F*_ST_ estimated between populations of continental origins (O'Brien & Moore 2000; Gonzalez *et al*. 2001; Novembre *et al*. 2005). This geographical cline has attracted the attention of several studies, and the *CCR5* variants have been proposed for involvement in several infections, including bubonic plague (Stephens *et al*. 1998), smallpox (Galvani & Slatkin 2003) and West Nile disease (Glass *et al*. 2006). The *Δ*32 mutation has been estimated to have occurred recently, between 700 and 5000 years ago (Stephens *et al*. 1998; Slatkin 2001; Hummel *et al*. 2005; Sabeti *et al*. 2005), and then to have increased rapidly in frequency because of its strong selective advantage (Libert *et al*. 1998; Stephens *et al*. 1998). The genealogy of *CCR5* haplotypes has deep branch lengths despite little differentiation among populations. Variation within the *CCR5* gene is much higher than expected and characterized by an excess of non-synonymous substitutions (less than 80%; Carrington *et al*. 1997, 1999). This finding suggested a deviation from neutrality not accounted for by population structure, which was confirmed by tests for natural selection (Bamshad *et al*. 2002).

Recently, Sabeti *et al*. (2005) concluded that while the possibility that some selection could not be ruled out at *CCR5*, the EHH estimates about *CCR5*-*Δ32* did not exceed neutral expectations. However, the *CCR5*-*Δ32*-bearing haplotype has been estimated by several authors to extend as far as 950–1000 kb or 60-fold longer than the HapMap average of 15 kb (Stephens *et al*. 1998; Bamshad *et al*. 2002; Sabeti *et al*. 2005; Frazer *et al*. 2007). Actually, the failure of the EHH test by Sabeti *et al.* (2005) is likely due to the occurrence of equally long adaptive *CCR-+*- (not the *CCR5*-*Δ32*)-bearing haplotypes, which diminish the *CCR5*-*Δ32*-bearing haplotypes’ apparent influence. There is extensive evidence for elevated d*N*/d*S* within *CCR5* in African and Asian populations, where *CCR5*-*Δ32* is absent, implying that alternative extended *CCR5*-+ haplotypes resulting from selection of different pathogens become evident (Carrington *et al*. 1997, 1999; Bamshad *et al*. 2002).

## 5. Human genome-wide scans for selection

Large human genotyping databases have been assembled (HapMap), and sequencing genomes of entire populations will soon become routine. As the amount of genome-wide SNP genotyping has accumulated, selection tests across human genomes have been attempted (table 3). One study represented comparative methods (Bustamante *et al*. 2005); four studies looked for gene neighbourhoods exhibiting extended LD (Huttley *et al*. 1999; Voight *et al*. 2006; Wang *et al*. 2006; Frazer *et al*. 2007); two studies looked for diminished polymorphism (Altshuler *et al*. 2005; Oleksyk *et al*. 2008); two studies looked for an aberrant frequency spectrum (Carlson *et al*. 2005; Nielsen *et al*. 2005); and two studies looked at the high values of local genomic divergence either alone (Akey *et al*. 2002), or in combination with diminished heterozygosity (Oleksyk *et al*. 2008). Finally, Tang *et al*. (2007) used admixture mapping in Puerto Ricans and found strong statistical evidence of recent selection in three chromosomal regions, including the human leucocyte antigen region on chromosome 6p (figure 7), chromosome 8q and chromosome 11q. Two of these regions harbour genes for olfactory receptors and all three exhibited deficiencies in the European-ancestry proportion.

**Table 3.** Comparison between GWSSs reported in 11 different studies.

study^a^	approaches used^b^	all sites	Europeans^c^		Africans^d^		Asians^e^	
		number reported	reported regions	replicated (hits)^f^	reported regions	replicated (hits)	reported regions	replicated (hits)
Huttley *et al*. (1999)	VII	10	10	9 (27)	—	—	—	—
Akey *et al*. (2002)	VI	153	141	15 (18)	105	9 (9)	43	5 (7)
Altshuler *et al*. (2005, HapMap)	IV, VI	213	72	25 (38)	75	24 (32)	87	23 (41)
Bustamante *et al*. (2005)	II	61	61	3 (3)	61	4 (4)	61	4 (4)
Carlson *et al*. (2005)	V	59	23	9 (19)	7	4 (11)	29	12 (28)
Nielsen *et al*. (2005)	V	23	23	9 (14)	23	2 (3)	—	—
Voight *et al*. (2006)	VII	776	256	49 (52)	271	37 (42)	249	43 (48)
Wang *et al*. (2006)	VII	117	117	19 (24)	117	14 (16)	117	11 (16)
Frazer *et al*. (2007, HapMap II)	VII	19	16	9 (27)	9	4 (4)	9	3 (4)
Sabeti *et al*. (2007)	VI, VII	42	23	9 (9)	—	—	22	9 (13)
Oleksyk *et al*. (2008)	IV, VI	179	161	36 (53)	102 (26)	10 (13)	(76)	8 (8)
total		1652	903	192 (284)	770	108 (134)	617	118 (169)

^a^Comparisons have been made by lifting genome coordinates for all the reported regions to that of hg18 (March 2006) using LiftOver executable within the UCSC genome browser. Gene coordinates were obtained by searching for their chromosome positions in NCBI using bioDBnet conversion tool (http://biodbnet.abcc.ncifcrf.gov).

^b^See table 1.

^c^European, European-American or the worldwide populations (local population not specified, or selection reported for two and more populations in the same region). Numbers of regions exclusive to each of the three populations are presented in the electronic supplementary material, figure S2.

^d^African, African-American or worldwide population.

^e^Asians or the worldwide population.

^f^The number outside of the parentheses represents the number of regions verified by other studies, while the number inside the parentheses represents the total number of times these regions have been verified by other studies. For example, Huttley *et al*. (1999) published 10 studies, nine of which were verified by a total of 27 different regions from 10 other genome-wide scans for selection.

## 6. A synthesis of scans across the genome

In table 3, we compared several scans to find sites of replication among different studies (see also Oleksyk *et al*. 2008). We adjusted for the locality of selection by subdividing putatively selected regions into three categories: (i) those discovered in European or European-American populations, (ii) those discovered in African or African-American populations, and (iii) those discovered in Asian populations. Comparisons between 11 selection scans in the three groups of populations are shown in table 3. A human genome map of overlapping sites, along with their coordinates, can be found in our earlier study (Oleksyk *et al*. 2008). Comparisons between studies have been attempted earlier, using gene names (Biswas & Akey 2006; Nielsen *et al*. 2007), but never by comparing coordinates among multiple GWSSs.

A comparison of 11 GWSSs using different datasets and methodologies provides a comprehensive summary of reported selection signatures across the genome. As different selection methods target different time periods, they can complement each other by pointing to different selection episodes during the evolutionary history of a species. Correspondingly, different scans that use similar methods should point to similar coordinates of selection regions. Scans should validate candidate genes that were discovered by similar methods. The analytical approaches to GWSSs described here also allow testing specific hypotheses involving candidate loci. So far, the coverage of candidate genes is modest. Of the 30 candidate genes previously reported to be selected (table 2), only nine (*LCT*, *CCR5*, *ADH1B*, *CYP3A5*, *FOXP2*, *MCPH1*, *DK5RAP2*, *SLC24A5* and *TTL.6*) were verified in one of the 11 GWSSs reviewed (table 2). Seven other genes (*HBB*, *CENPJ*, *FY*, *Il13*, *Il4*, *HFE* and *TRPV6*) were within 200 kb from one selected region. Remarkably, only two of these gene regions were verified by two or more studies (*LCT* and *CYP3A5*), and four more were positioned within a selected region in one study, but less than 200 kb away from at least one region in other GWSSs (*CCR5*, *ADH1B* and *SLC24A5*; table 2).

Finding a candidate gene using one of the tests (table 1) does not assure that it will be found in the GWSS, even if the GWSS incorporates the same test used in the initial analysis of the selection signature. For instance, *G6PD* and *TNSF5* genes have been shown to be under a strong selection in Africans (Sabeti *et al*. 2002), but did not make the list of selected regions found in the GWSS by the same EHH methodology (Altshuler *et al*. 2005; Frazer *et al*. 2007; Sabeti *et al*. 2007) (table 2). Similarly, long haplotypes around a rare *CCR5*-*Δ32* deletion in *CCR5* have been shown to be a more common feature in the genome than was previously thought (Sabeti *et al*. 2005). This can be explained either by the insufficient power of the tests employed, or by the insufficient coverage in the scanned datasets; or this may indicate their relatively modest selective effect, compared with the other candidate genes included in the list (Sabeti *et al*. 2006). Similarly, the *LCT* gene that has become a hallmark of recent selection testing (Bersaglieri *et al*. 2004; Nielsen *et al*. 2005; Voight *et al*. 2006) has not been found by other studies (Huttley *et al*. 1999; Akey *et al*. 2002; Altshuler *et al*. 2005; Bustamante *et al*. 2005; Carlson *et al*. 2005; Nielsen *et al*. 2005; Voight *et al*. 2006; Wang *et al*. 2006; Oleksyk *et al*. 2008).

Historically, most of the candidate regions in the list were discovered by methods that identify older selection (table 1, I–V). Methodology for detecting recent selection has improved in the recent decade, specifically by incorporating LD methods (Sabeti *et al*. 2002; Voight *et al*. 2006; Wang *et al*. 2006). As the number of dense genotyped sets increases with improved genotyping technology and next-generation sequencing, we should see an increased precision of selection events documented. These new GWSSs should incorporate a multi-layer approach by including several tests capturing maximum information from different selection signatures. Bottlenecks and population expansion create a problem for other methods: they alter LD pattern and frequency spectrum, reduce heterozygosity and change admixture contribution. However, as most of the GWSSs include hundreds of thousands of loci, and as demographic events impact loci genome-wide, it is possible to account for genome-wide effects by comparing regional statistics directly.

## 7. Conclusions

We have attempted in this review to summarize the new approaches, findings and implications of genome GWSSs to probe for perturbations that result from selective episodes that afflicted our ancestors. Though theoretically appealing, a puzzlement arises when we inspect how modest is the replication for discovery of different genomic regions between algorithmic approaches or between different studies (tables 2 and 3). Several possible explanations contribute to this disconnect, but two are worth mentioning. First, as even the strongest strong selective episodes are temporary, the entropy of subsequent mutational/ recombination events rapidly diminish the intensity of selective footprints for which we search. As genomic selection footprints decay at different rates for different algorithms, a negative result does not necessarily mean that selection did not happen there. Second, there are likely false-positive signals that do not reflect historic selection at all; rather they arise from local genomic differences in DNA repair, mutation rate differential, recombination difference, sequence stability, and the statistical outlier effects of multiple genome-wide tests for significance. Nonetheless, as we scroll though DNA sequences of human and available mammals (Lewin *et al*. in press), we are beginning to uncover signals that make sense (see examples in §3*a*–*e*), ones that we can interpret in the context of human history, culture, geography and archaeology. In some ways, these imputations will preview similar creative approaches to connecting gene organization in a holistic systems biology context, ones that promise to inform life scientists of how genome codes specify individual and species development and one day soon nearly all things biological. Genome sequences of non-traditional species will quickly appear with the advancing faster and cheaper next-generation sequencing technologies projecting some 10 000 vertebrate species genome sequences assessed in the next decade (G1KCOS in press). With these available genome sequences complemented by powerful informatics routines to assemble and annotate the data, numerous anticipated discoveries will be revealed in both the comparative and population diversity context in a way that expands biological enquiry in dimensions across geographical populations, among related species, to higher taxa, and, importantly, back though the formative evolutionary history of humankind and those modern species with which we share our planet.
